# Supplementary material for: Splice-Junction-Based Mapping of Alternative Isoforms in the Human Proteome
Source: Cell Rep. Author manuscript; Available in PMC 2020 Jan 15. (PMC6961840; doi:10.1016/j.celrep.2019.11.026)
Supplement: 2 [file NIHMS1546469-supplement-2.zip › DF1/PXD006675/RightVentricle/RightVentricle_30_CCS_GSGQLRVQDVEVHLEDQMVLVHTTLPSQEVQALLEGTGR.pdf]

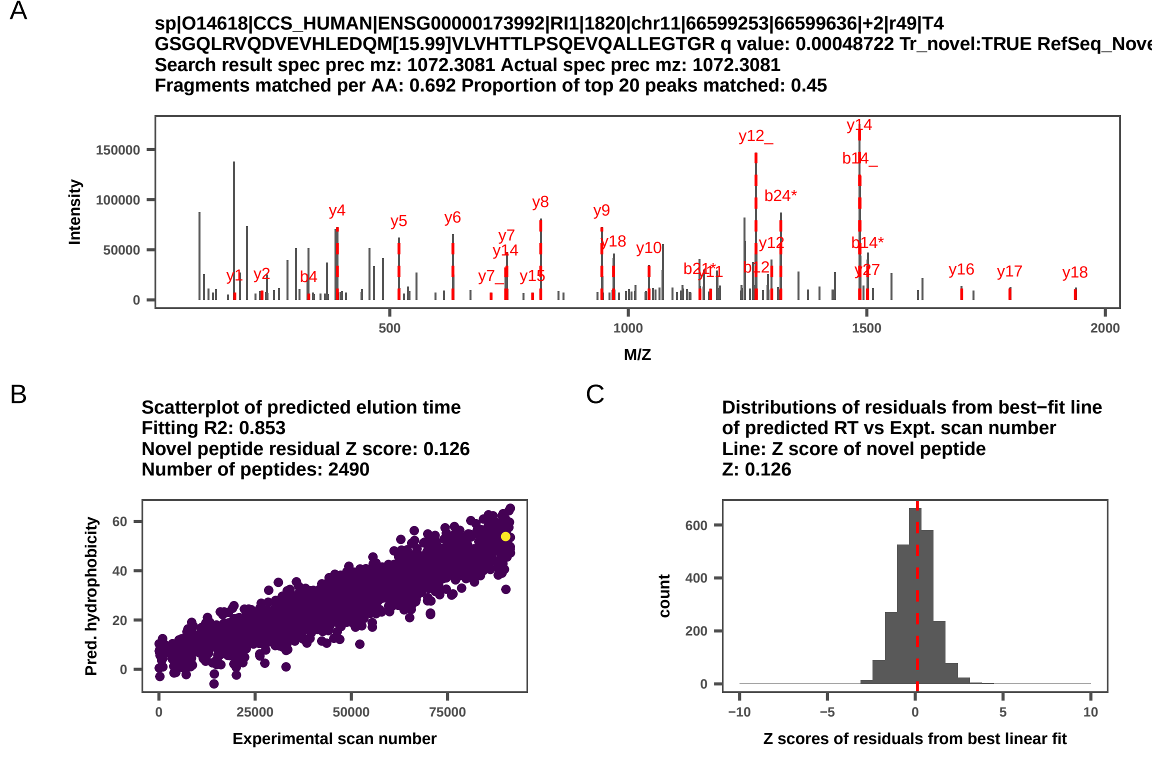
A
sp|O14618|CCS_HUMAN|ENSG00000173992|RI1|1820|chr11|66599253|66599636|+2|r49|T4
GSGQLRVQDVEVHLEDQM[15.99]VLVHTTLPSQEVQALLEGTGR q value: 0.00048722 Tr_novel:TRUE RefSeq_Nove
Search result spec prec mz: 1072.3081 Actual spec prec mz: 1072.3081
Fragments matched per AA: 0.692 Proportion of top 20 peaks matched: 0.45
Intensity
150000
100000
50000
0
y14
y12_
b14_
b24*
y4
y5
y6
y8
y9
y7
y14
y18
y10
y12
b14*
y1
y2
b4
y7_
y15
b21*
y11
b12
y27
y16
y17
y18
500
1000
1500
2000
M/Z
B
Scatterplot of predicted elution time
Fitting R2: 0.853
Novel peptide residual Z score: 0.126
Number of peptides: 2490
Pred. hydrophobicity
60
40
20
0
0
25000
50000
75000
Experimental scan number
C
Distributions of residuals from best−fit line
of predicted RT vs Expt. scan number
Line: Z score of novel peptide
Z: 0.126
count
600
400
200
0
−10
−5
0
5
10
Z scores of residuals from best linear fit
